# SENIOR CENTER RESPONSE TO COVID-19: INVOLVEMENT WITH VACCINE DISTRIBUTION

**DOI:** 10.1093/geroni/igac059.1790

**Published:** 2022-12-20

**Authors:** Ceara Somerville, Caitlin Coyle, Jan Mutchler

**Affiliations:** University of Massachusetts Boston, Boston, Massachusetts, United States; University of Massachusetts Boston, Boston, Massachusetts, United States; University of Massachusetts Boston, Boston, Massachusetts, United States

## Abstract

In early 2021, access to a COVID-19 vaccine was prioritized for older adults and people with multiple co-morbidities. Between high demand, emerging supply, and new systems for booking a vaccine appointment, many people had challenges getting an appointment. Senior centers became a crucial resource for access to the vaccine and additional information about its efficacy and safety. This poster presents survey data collected from 282 senior centers in Massachusetts regarding their involvement with the COVID-19 vaccine distribution in 2021. Nearly all senior centers reported making vaccine appointments on behalf of residents and were responsible for over 166,000 appointments made. Nearly 100,000 hours were spent by senior center staff or volunteers to make appointments. About a third of senior centers participated in hosting nearly 2,000 vaccine clinics, which vaccinated over 175,000 adults. Respondents reported challenges faced during the booking process, including the length of time and the developing technologies to book an appointment. Almost two-thirds of senior centers reported assisting community members under age 60, operating beyond the traditional scope of their services. Other assistance provided by senior centers included providing information about eligibility and guidelines (84%), information about vaccine efficacy and safety (62%), offering transportation to appointments (70%), and providing physical assistance for appointments (31%). Evidence from this poster emphasizes the important role that senior centers play in the community, not just for older adults but also for the community at large.

